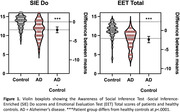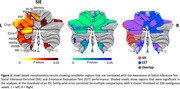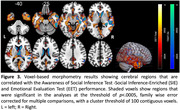# Cerebellar and cerebral contributions to social inference impairments in early stages of Alzheimer’s disease

**DOI:** 10.1002/alz.091928

**Published:** 2025-01-03

**Authors:** Yu Chen, Bailey A. McEachen, Faatimah Syed, Rea Antoniou, Myrthe Gwen Rijpma, Howard J. Rosen, Joel H. Kramer, Bruce L. Miller, Katherine P. Rankin

**Affiliations:** ^1^ University of California San Francisco, San Francisco, CA USA

## Abstract

**Background:**

Despite the prevalent belief that socioemotional processing remains mostly intact in the early stages of Alzheimer’s disease (AD), impaired Theory of Mind – the ability to understand and interpret others’ thoughts, beliefs, and feelings – has been observed in persons with AD. During everyday conversations, the high cognitive loading of socioemotional interactions may adversely impact the ability of persons with AD to identify emotions and read others’ intentions. This study aimed to investigate socioemotional perception capabilities in early‐stage AD and to determine whether cerebellar and cerebral integrity predicts performance on socioemotional perception tests.

**Method:**

Fifty‐two individuals meeting McKhann criteria for probable AD syndrome and 52 healthy older controls were evaluated with the Awareness of Social Inference Test (TASIT) – Emotional Evaluation Test (EET) and Social Inference‐Enriched (SIE). These tests use videos of conversations to evaluate the ability to identify basic emotions and understand intentions in realistic everyday scenarios. The EET evaluates emotion reading and the SIE examines social inference understanding. We further conducted voxel‐based morphometry with participants’ earliest structural magnetic resonance imaging scans to identify the relationship of cerebellar and cerebral integrity with individual task performance (*p*<.05 for cerebellar, *p*<.0005 for cerebral associations, family wise error corrected).

**Results:**

Patients with very early‐stage AD (Global Clinical Dementia Rating = 0.8±0.4) performed worse on both TASIT‐EET and TASIT‐SIE relative to healthy controls (*p*<.05). Subregions of the cerebellum including bilateral lobules I‐VI, VIIIa, VIIIb, IV, Crus I, II, and the vermis were associated with TASIT‐EET performance. The entire left cerebellar hemisphere, right lobules VI, VIIIa, IX, Crus I, and the vermis were implicated in TASIT‐SIE performance. Cortical subregions in networks involved in memory, semantic comprehension, salience, and socioemotional interpretation were associated with TASIT performance, with TASIT‐SIE more right lateralized.

**Conclusion:**

These results clarify that in early‐stage AD, cerebellar involvement and its interaction with corresponding cerebral regions may play a more important role in socioemotional processing than previously appreciated. Specifically, left‐lateralized cerebellar and right‐lateralized cerebral damage predicted poorer social inference understanding in persons with AD. Their emotion reading, in contrast, involved cerebellar and cerebral hemispheres more bilaterally.